# Detection of Important Scenes in Baseball Videos via a Time-Lag-Aware Multimodal Variational Autoencoder †

**DOI:** 10.3390/s21062045

**Published:** 2021-03-14

**Authors:** Kaito Hirasawa, Keisuke Maeda, Takahiro Ogawa, Miki Haseyama

**Affiliations:** 1 Graduate School of Information Science and Technology, Hokkaido University, N-14, W-9, Kita-ku, Sapporo 060-0814, Hokkaido, Japan; 2 Office of Institutional Research, Hokkaido University, N-8, W-5, Kita-ku, Sapporo 060-0808, Hokkaido, Japan; maeda@lmd.ist.hokudai.ac.jp; 3 Faculty of Information Science and Technology, Hokkaido University, N-14, W-9, Kita-ku, Sapporo 060-0814, Hokkaido, Japan; ogawa@lmd.ist.hokudai.ac.jp (T.O.); miki@ist.hokudai.ac.jp (M.H.)

**Keywords:** multimodal variational autoencoder, detection of important scenes, Twitter, sports video, time-lags

## Abstract

A new method for the detection of important scenes in baseball videos via a time-lag-aware multimodal variational autoencoder (Tl-MVAE) is presented in this paper. Tl-MVAE estimates latent features calculated from tweet, video, and audio features extracted from tweets and videos. Then, important scenes are detected by estimating the probability of the scene being important from estimated latent features. It should be noted that there exist time-lags between tweets posted by users and videos. To consider the time-lags between tweet features and other features calculated from corresponding multiple previous events, the feature transformation based on feature correlation considering such time-lags is newly introduced to the encoder in MVAE in the proposed method. This is the biggest contribution of the Tl-MVAE. Experimental results obtained from actual baseball videos and their corresponding tweets show the effectiveness of the proposed method.

## 1. Introduction

The development of various network technologies and devices has resulted in the availability of many video distribution services. A tremendous number of videos has become viewable via these services. Since sports videos are popular videos among them, a large number of sports games is provided by sports video distribution services. Although users can easily obtain their desired videos, it is difficult for viewers to watch all of the games provided by these services [1]. Among various sports, a baseball game takes a longer time than other sports such as basketball and soccer. Therefore, techniques for viewers to understand the content of a long game in a short time are necessary [1].

The generation of highlights is one of the important techniques for enabling a long game to be watched in a short time. Highlights consist of important scenes such as scoring and home runs. Therefore, since important scene detection methods are necessary for the generation of highlights, various detection methods have been proposed [2,3,4,5,6,7,8]. The methods in [2,3,4] using videos detect important scenes by applying a hidden Markov model and a maximum entropy model to player movements and cheers from the audiences obtained from the target videos. Therefore, conventional video-based methods are effective for the representation of visual information of players and audiences. On the other hand, along with the development of microblogging services, methods using Twitter (https://twitter.com/ (accessed on 12 March 2021) for the detection of important scenes have been proposed [5,6,7]. By using Twitter, which is one of the microblogging services, the reactions of viewers during games can be obtained. People watching baseball games often post tweets, which are short text messages, related to games. Since posted tweets often include information on the reactions of viewers and the contents of the games, conventional Twitter-based methods have an advantage for the extraction of opinions or feelings of viewers. Therefore, by taking advantage of each modality, it is expected that a method using both Twitter and video analysis would enable high-quality detection of important scenes.

In order to use both tweets and videos, we need to consider multiple different modalities. Several methods focusing on the relationships across multiple different modalities have been proposed [9,10,11]. The method in [9] realizes efficient cross-modal video-text retrieval using multi-modal features such as visual characteristics, audio, and text. Then, the method in [10] learns multimodal embeddings between modalities such as text, video, and audio via deep canonical correlation analysis. The method in [11] has a multimodal variational autoencoder (MVAE) [12] including a fake news detection network using tweets and visual information, and the performance has been improved. Thus, MVAE is effective for our task, which is the detection of important scenes using tweets and videos. Generally, the MVAE can detect correlations across modalities by training shared representations across them. Since the MVAE can flexibly express the relationship between heterogeneous modalities, the construction of a highly accurate method for our tasks is expected by adopting the MVAE. However, to efficiently use tweets and videos, we need to consider the following problem. Since tweets posted on Twitter are influenced by multiple previous events in the videos, they are closely related to each other. Thus, there are time-lags between posted tweets and multiple corresponding events. Since these time-lags cannot be considered in previous MVAE-based approaches, the development of the MVAE-based detection method considering time-lags between posted tweets and multiple corresponding events would contribute to an improvement in performance.

In this paper, we propose a new method for the detection of important scenes in baseball videos via the MVAE considering time-lags between tweets and corresponding multiple previous events. The newly proposed method is the time-lag-aware MVAE (Tl-MVAE). To realize the detection using tweets and videos, tweet, video, and audio features extracted from tweets and videos are used in the Tl-MVAE. The Tl-MVAE consists of an encoder, a decoder, and an important scene detector. In the encoding architecture, multimodal features are transformed into latent features based on feature correlations considering time-lags. The decoder reconstructs the original transformed features from the latent features. Moreover, the important scene detector detects important scenes by using the latent features. Since feature transformation based on feature correlations considering such time-lags is introduced to the Tl-MVAE, the proposed method can derive latent features that are efficient for the consideration of the relationships between tweets and videos. From this novelty, the proposed method can realize accurate detection based on the Tl-MVAE using multimodal features extracted from tweets and videos.

It should be noted that this paper is an extended version of [13]. Specifically, we newly introduce the novel Tl-MVAE to the detection of important scenes.

## 2. Detection of Important Scenes via the TL-MVAE

**Figure 1:**
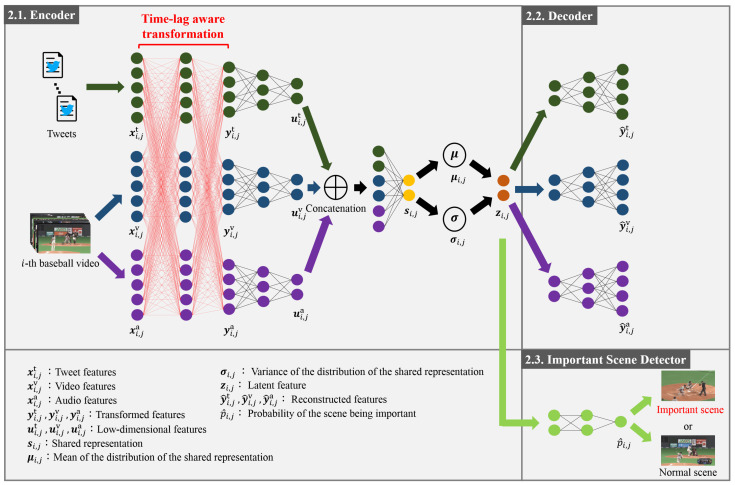
Outline of the time-lag-aware multimodal variational autoencoder (Tl-MVAE). The Tl-MVAE encodes tweet, video, and audio features into latent features based on feature correlations with the consideration of time-lags, as described in Section 2.1. Furthermore, the Tl-MVAE reconstructs the original features from the latent features as described in Section 2.2. Finally, the Tl-MVAE detects important scenes by using the latent features as described in Section 2.3.

The details of the new method for the detection of important scenes in baseball videos via the Tl-MVAE are shown in this section. As shown in Figure 1, the Tl-MVAE consists of an encoder (Section 2.1), a decoder (Section 2.2), and an important scene detector (Section 2.3). Each of them is explained in detail below. The final loss and the optimization of the parameters of the encoder, decoder, and important scene detector are also shown in Section 2.4.

### 2.1. Encoder

The encoder is explained in this subsection. The inputs of the encoder are tweets posted during baseball games and videos, and the outputs are the latent features of the features extracted from tweets and videos. Specifically, in feature extraction architectures, by using the *i*-th baseball video (i=1,2,…,I; *I* being the number of videos) and corresponding tweets, features $x_{i,j}^{m}\in R^{d_{x}}(j=1,2,\ldots ,J_{i};J_{i}$ being the number of tweets of the *i*-th video) are calculated. Note that m∈{t,v,a} is the modality in the feature extraction architecture. Consequently, feature matrices $X_{i}^{m}=\left[x_{i,1}^{m},\ldots ,x_{i,j}^{m},\ldots ,x_{i,J_{i}}^{m}\right]\in R^{d_{x}\times J_{i}}$ are obtained. The feature extraction architectures are explained in detail in Section 3.1.

**Figure 2:**
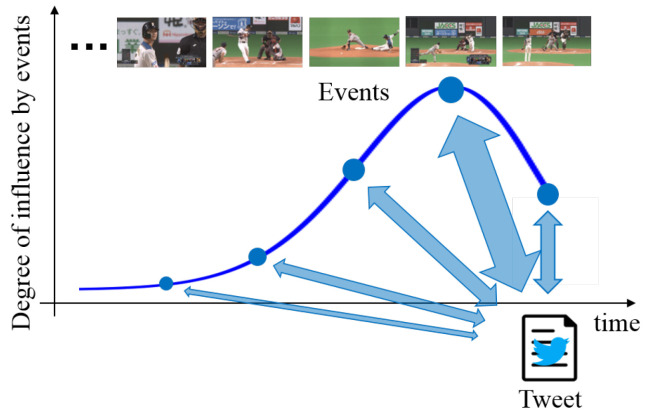
The relationship between a tweet and its corresponding multiple previous events. The tweet corresponds to events from the present to the past. Furthermore, these events are weighted by using the degree of influence defined on the basis of the Poisson distribution.

The proposed network transforms extracted features with the consideration of time-lags between tweets and videos. Specifically, by transforming feature matrices $X_{i}^{m}$ via multi-layered neural networks, $Y_{i}^{m}=\left[y_{i,1}^{m},\ldots ,y_{i,j}^{m},\ldots ,y_{i,J_{i}}^{m}\right]\in R^{d_{y}\times J_{i}}$ considering time-lags are output from the last layer of the neural network. Since posted tweets are related to corresponding multiple previous events rather than just one event, there exists a correlation between tweet features at a target time and corresponding video and audio features of multiple previous events. For example, the immediate previous event strongly influences the tweet, and the influence of past events tends to be gradually weakened. Thus, we assume that tweets are affected by events from the present to the past according to a Poisson distribution, which means that the number of events occurring in a fixed interval of time can be regarded as the degree of influence by baseball events as shown in Figure 2. From this assumption, we construct multi-layered neural networks with consideration of the time-lags between different features based on the influence defined from the Poisson distribution. In order to calculate $Y_{i}^{m}$ considering these time-lags with the optimization of the parameters of the multi-layered neural networks, we maximize the average inter-set correlation [14] defined as:

(1) $$\rho =\frac{1}{d_{y}\left(M-1\right)}\sum_{d=1}^{d_{y}}\frac{\psi_{d}^{⊤}R_{B}\psi_{d}}{\psi_{d}^{⊤}R_{W}\psi_{d}},$$

where $\psi_{d}\in R^{d_{y}}\left(d=1,2,...,d_{y}\right)$ means the optimal weight common to all modalities. Furthermore, M(=3) means the number of modalities. Then, $R_{B}$ is the between-set covariance matrix considering the time-lags, and $R_{W}$ is the within-set covariance matrix. They are respectively defined as:

(2) $$\begin{array}{cc} R_{B} & =\sum_{i=1}^{I}\;\sum_{m_{1}\in \left\{t,v,a\right\}}\;\sum_{m_{2}\in \left\{t,v,a\right\},m_{2}\neq m_{1}}{\bar{C}}_{i}^{m_{1},m_{2}}, \end{array}$$

(3) $$\begin{array}{cc} R_{W} & =\sum_{i=1}^{I}\;\sum_{m\in \{t,v,a\}}{\underline{C}}_{i}^{m,m}. \end{array}$$

Note that we omit the same scaling value ${\left(J_{i}-1\right)}^{-1}M^{-1}$ in the above equations. Furthermore,

(4) $${\bar{C}}_{i}^{m_{1},m_{2}}=\left\{\begin{array}{cc} \; & \frac{\sum_{l=0}^{L-1}\frac{e^{-\lambda }\lambda^{l}}{l!}{\tilde{Y}}_{i,0}^{m_{1}}{\tilde{Y}}_{i,l}^{m_{2}⊤}}{\sum_{l=0}^{L-1}\frac{e^{-\lambda }\lambda^{l}}{l!}}\;\left(m_{1}\in \left\{t\right\},m_{2}\in \left\{v,a\right\}\right)\\ \; & \frac{\sum_{l=0}^{L-1}\frac{e^{-\lambda }\lambda^{l}}{l!}{\tilde{Y}}_{i,l}^{m_{1}}{\tilde{Y}}_{i,0}^{m_{2}⊤}}{\sum_{l=0}^{L-1}\frac{e^{-\lambda }\lambda^{l}}{l!}}\;\left(m_{1}\in \left\{v,a\right\},m_{2}\in \left\{t\right\}\right)\\ \; & {\tilde{Y}}_{i,0}^{m_{1}}{\tilde{Y}}_{i,0}^{m_{2}⊤}\;\left(\mathrm{otherwise}\right) \end{array}\right.,$$

(5) $${\underline{C}}_{i}^{m,m}={\tilde{Y}}_{i,0}^{m}{\tilde{Y}}_{i,0}^{m⊤},$$

where *L* determines the number of previous events affecting the target tweet. In addition, λ is a parameter of the Poisson distribution that controls the peak of the distribution. Note that λ is the same as the mean and the variance of the distribution. Then, feature matrices ${\tilde{Y}}_{i,l}^{m}=\left[y_{i,L-l}^{m},\ldots ,y_{i,J_{i}-l}^{m}\right]\;\left(l=0,\ldots ,L-1\right)$ are mean-normalized.

Finally, we calculate latent features from the transformed features $y_{i,j}^{m}$. Specifically, we pass $y_{i,j}^{m}$ through multiple fully connected layers and obtain low-dimensional features $u_{i,j}^{m}$. The low-dimensional tweet features $u_{i,j}^{t}$, video features $u_{i,j}^{v}$, and audio features $u_{i,j}^{a}$ are concatenated and passed through a fully connected layer to form the shared representation $s_{i,j}$. Then, the mean $\mu_{i,j}$ and the variance $\sigma_{i,j}$ of the distribution of the shared representation $s_{i,j}$ are obtained. By using random variables ϵ sampled from the Gaussian distribution, the latent features $z_{i,j}$ are defined as:

(6) $$z_{i,j}=\mu_{i,j}+\sigma_{i,j}\circ \epsilon .$$

By denoting the network that outputs the latent features $z_{i,j}$ from the transformed features $y_{i,j}^{m}$ as $G_{\mathrm{enc}}\left(y_{i,j}^{m},\theta_{\mathrm{enc}}\right)$, the latent features $z_{i,j}$ are denoted as:

(7) $$z_{i,j}=G_{\mathrm{enc}}\left(y_{i,j}^{m},\theta_{\mathrm{enc}}\right),$$

where $\theta_{\mathrm{enc}}$ means all of the parameters to be trained in the network that outputs the latent features $z_{i,j}$ from the transformed features $y_{i,j}^{m}$.

Introducing the consideration of time-lags between tweets and corresponding multiple previous events into the encoder is the biggest contribution. The encoder of a general MVAE cannot consider time-lags between different modalities. However, the Tl-MVAE can flexibly consider the characteristics of tweets and videos by the consideration of time-lags based on Equation (4).

### 2.2. Decoder

In this subsection, we explain the decoder that reconstructs the original data from the latent features. The decoder is an inverted network of the network that outputs the latent features from the transformed features. Specifically, the input of the decoder is the latent features $z_{i,j}$ obtained in Section 2.1, and it outputs reconstructed features ${\hat{y}}_{i,j}^{m}$. By denoting the decoder as $G_{\mathrm{dec}}\left(z_{i,j},\theta_{\mathrm{dec}}\right)$, the reconstructed features are denoted as:

(8) $${\hat{y}}_{i,j}^{m}=G_{\mathrm{dec}}\left(z_{i,j},\theta_{\mathrm{dec}}\right),$$

where $\theta_{\mathrm{dec}}$ means all of the parameters to be trained in the decoder.

### 2.3. Important Scene Detector

In this subsection, we explain the important scene detector consisting of multiple fully connected layers. The important scene detector takes the latent features obtained in Section 2.1 as the input and aims to classify the scene as important or normal. Specifically, latent features $z_{i,j}$ are input, and the probability ${\hat{p}}_{i,j}$ of the scene corresponding to $z_{i,j}$ being important is output. By denoting the important scene detector as $G_{\mathrm{isd}}\left(z_{i,j},\theta_{\mathrm{isd}}\right)$, the probability ${\hat{p}}_{i,j}$ is defined as:

(9) $${\hat{p}}_{i,j}=G_{\mathrm{isd}}\left(z_{i,j},\theta_{\mathrm{isd}}\right),$$

where $\theta_{\mathrm{isd}}$ means all of the parameters to be trained in the important scene detector. When ${\hat{p}}_{i,j}>\tau$, the scene is determined to be an important scene. Note that we predetermine a threshold value τ.

### 2.4. Final Loss

In this subsection, we explain the final loss of the Tl-MVAE. We jointly train the encoder, decoder, and important scene detector. Thus, the final loss can be defined as:

(10) $$L_{\mathrm{final}}\left(\theta_{\mathrm{enc}},\theta_{\mathrm{dec}},\theta_{\mathrm{isd}}\right)=(\sum_{m\in \{t,v,a\}}\xi_{\mathrm{rec}}^{m}L_{\mathrm{rec}}^{m})+\xi_{\mathrm{kl}}L_{\mathrm{kl}}+\xi_{\mathrm{isd}}L_{\mathrm{isd}},$$

where $L_{\mathrm{rec}}^{m}$ is the reconstruction loss between the outputs of the decoder and features considering time-lags obtained in the encoder. The reconstruction loss $L_{\mathrm{rec}}^{m}$ is defined as:

(11) $$L_{\mathrm{rec}}^{m}=\frac{1}{I}\sum_{i=1}^{I}\{\frac{1}{J_{i}d_{y}}\sum_{j=1}^{J_{i}}\sum_{d=1}^{d_{y}}{\left({\hat{y}}_{i,j,d}^{m}-y_{i,j,d}^{m}\right)}^{2}\}.$$

Note that ${\hat{y}}_{i,j,d}^{m}$ and $y_{i,j,d}^{m}$ are the *d*-th values of ${\hat{y}}_{i,j}^{m}$ and $y_{i,j}^{m}$, respectively. Then, the minimization of the KL divergence $L_{\mathrm{kl}}$ means optimizing the probability distribution parameters (μ and σ) to closely resemble those of the target distribution (Gaussian distribution). The KL divergence $L_{\mathrm{kl}}$ is defined as:

(12) $$L_{\mathrm{kl}}=\frac{1}{I}\sum_{i=1}^{I}\{\frac{1}{2J_{i}}\sum_{j=1}^{J_{i}}\sum_{d=1}^{d_{z}}\left(\mu_{i,j,d}^{2}+\sigma_{i,j,d}^{2}-\log\left(\sigma_{i,j,d}\right)-1\right)\},$$

where $\mu_{i,j,d}$ and $\sigma_{i,j,d}$ are the *d*-th values of $\mu_{i,j}$ and $\sigma_{i,j}$, respectively. Moreover, $L_{\mathrm{isd}}$, which is the detection loss of important scenes, is defined as:

(13) $$L_{\mathrm{isd}}=-\frac{1}{I}\sum_{i=1}^{I}\{\frac{1}{J_{i}}\sum_{j=1}^{J_{i}}\left(l_{i,j}\log\left({\hat{l}}_{i,j}\right)+\left(1-l_{i,j}\right)\log\left(1-{\hat{l}}_{i,j}\right)\right)\},$$

where ${\hat{l}}_{i,j}$ is the label calculated on the basis of the probability ${\hat{p}}_{i,j}$ and $l_{i,j}$ is the ground truth label. Then, $\xi_{\mathrm{rec}}^{m}$, $\xi_{\mathrm{kl}}$, and $\xi_{\mathrm{isd}}$ are parameters to balance the individual terms of the loss function. By minimizing the final loss, we can calculate the optimal parameters.

Our biggest contribution is the development of a method for the detection of important scenes in baseball videos via the Tl-MVAE, which can consider time-lags between tweets and their corresponding multiple previous events. The Tl-MVAE assumes that tweet features are related to the previous video and audio features, and feature transformation considering these characteristics is introduced into the encoder of the Tl-MVAE. Then, by using the latent features calculated from the features transformed on the basis of feature correlations considering time-lags, the accurate detection of important scenes can be realized. This is the novel idea of the Tl-MVAE.

## 3. Experimental Results

### 3.1. Experimental Setting

As datasets, twelve baseball videos (30 fps) and tweets posted during those games were used. The baseball videos were broadcast from 13 June to 27 September 2019 by Pacific League TV according to our previous experiments [13,15]. The details of these videos are shown in Table 1. By using the query “#lovefighters”, which is an official hashtag of the professional baseball team, we collected tweets for this experiment. We used seven games for the training of the Tl-MVAE and the other five games for the test. The previous works [3] and [4] used 6 and 10 games for the experiments. Moreover, since the proposed method adopts not only videos, but also tweets, more information is available in the proposed method than in the previous works. Thus, the number of games used in this experiment is considered to be sufficient. Furthermore, since tweets related to each experiment were collected on their own in previous works [7,8], we also followed their schemes in the same manner and verified the performance of the Tl-MVAE in this experiment. In the feature extraction architecture, as the tweet feature extraction networks described in Section 2.1, we adopted Tweet2Vec [16], which is one of the common language models. Tweet2Vec contains a bi-directional gated recurrent unit [17], a linear layer, and a softmax layer. Tweet2Vec used in the Tl-MVAE was trained by using tweets collected on the basis of 27 hashtags related to professional baseball teams. In order to extract the video features, we used 3D ResNet [18], which was pre-trained on the Kinetics dataset [19], as the video feature extraction network described in Section 2.1. 3D ResNet includes 17 convolutional blocks, a global average pooling layer, a fully connected layer, and a softmax layer. In addition, VGG16 [20], which was pre-trained with the ImageNet dataset [21], was used as the audio feature extraction network described in Section 2.1. VGG16 consists of 13 convolutional layers, five max layers, three fully connected layers, and a softmax layer. Generally, VGG16 is used for feature extraction from images. However, since spectrogram-based feature calculation based on a pre-trained CNN model leads to effective representation of audio data [22,23], we extracted audio features from spectrograms of audio based on the pretrained VGG16. Furthermore, $d_{x}$, $d_{y}$, *L*, λ$\xi_{\mathrm{rec}}^{m}$, $\xi_{\mathrm{kl}}$, $\xi_{\mathrm{isd}}$, and τ were set to 500, 256, 12, 3, 1, 1, 1, and 0.5, respectively. $d_{x}$, $d_{y}$, *L*, λ, and τ were set to the values at which the proposed method had the highest specificity. In addition, $\xi_{\mathrm{rec}}^{m}$, $\xi_{\mathrm{kl}}$, and $\xi_{\mathrm{isd}}$ were set to 1 according to [11].

In order to confirm the validity of introducing the tweet, video, and audio features, the following comparative methods (CMs1-7) were adopted. In addition, we adopted CMs8 and 9 to confirm the validity of the consideration of time-lags between tweets and corresponding multiple previous events. Furthermore, CMs10 and 11 were used to confirm the validity of using the MVAE as the model for the detection of important scenes. We used CM12 for the comparison with our previous method.

CMs1-6: Methods using features shown in Table 2. Note that CMs4 and 5 consider time-lags.

CM7: A method based on the simple integration of detection models constructed for each modality. This approach determines important scenes by majority voting of the detection results based on CMs1-3.

CM8: A method not considering time-lags based on the MVAE [11].

CM9: A method based on [8]. This approach considers time-lags between tweets and only one corresponding previous event.

CM10: A method [15] that flexibly expresses the relationship between tweets and videos based on time-lag-aware deep multiset canonical correlation analysis (Tl-dMCCA). This approach considers time-lags between tweets and corresponding multiple previous events.

CM11: A method using a long short-term memory (LSTM) [24], which can maintain a long-term memory, which is effective for training time series features calculated from tweets and videos. In the same manner as the Tl-MVAE, the inputs are tweets and videos, and the outputs are the probabilities that the scenes are important. This approach can consider time-lags.

CM12: A method using LSTM based on our previous method [13]. The inputs of LSTM are features transformed based on dMCCA, and the outputs are the probabilities that the scenes are important. This approach cannot consider time-lags.

In this experiment, each at-bat (an at-bat is a period from when the batter enters the batter box until he/she is out or reaches the bases) was regarded as an important scene when at least 80% of its length is detected as important. Since the specificity of the proposed method is the highest when this percentage of length is 80%, we adopted this percentage. As a quantitative evaluation index, we adopted specificity when sensitivity was almost 1.0 (i.e., maximizing sensitivity). Specificity when sensitivity is almost 1.0 means how much over-detection of normal scenes can be reduced when important scenes are detected. The detection of all important scenes is needed to maximize sensitivity. Thus, the over-detection of normal scenes needs to be close to 0.0 to obtain high specificity when sensitivity is almost 1.0. Since that is a difficult task, the specificity when sensitivity is almost 1.0 does not become a very large value in this experiment. Note that we defined pre-edited highlights broadcast by Pacific League TV as important scenes, i.e., ground truth. Other scenes are defined as normal scenes.

### 3.2. Performance Evaluation

The specificity of the proposed method (PM) and CMs1-12 are shown in Table 3. Since the PM has higher specificity than the specificity of CMs1-6, we can confirm the effectiveness of introducing tweet, video, and audio features. Furthermore, since the specificity of the PM is higher than that of CM7, simply utilizing these features does not necessarily improve detection performance. Thus, we can see that the consideration of the relationships between tweets and videos is effective for the detection of important scenes. Since the specificity of CM9 is higher than that of CM8, the effectiveness of the consideration of time-lags can be confirmed. When the t-test was performed on the probabilities calculated by CM8 and CM9, the *p*-value was 0.046, so there is a significant difference in this result. Furthermore, the PM has higher specificity than that of CM9. However, since the results of the PM and CM9 are very close, we compared these two methods by using the F-measure, which is the harmonic mean of recall and precision. As a result, the F-measure of the PM and CM9 were 0.416 and 0.404. Thus, we can confirm that the consideration of time-lags between tweets and corresponding multiple previous events is effective. Since the PM outperforms CMs10 and 11, feature transformation considering time-lags is effective for the detection of important scenes. In addition, the PM has higher specificity than that of CM12, which is our previous method. From the results of the PM and CMs1-12, accurate detection of important scenes of baseball videos via the Tl-MVAE considering time-lags between tweets and corresponding multiple previous events is realized.

**Figure 3:**
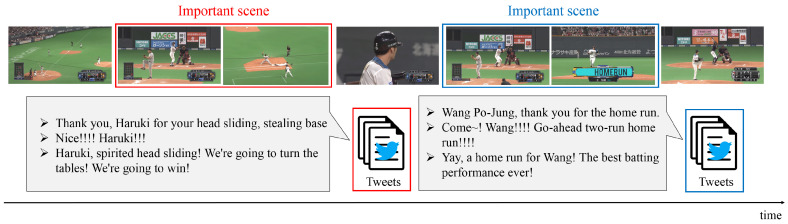
Two examples of important scenes detected by the Tl-MVAE and tweets corresponding to these scenes. The horizontal axis means time. The timing when important scenes occur and the timing when the corresponding tweets are posted are different.

Next, two examples of important scenes detected by our method (PM) and their corresponding tweets are shown in Figure 3. The tweets correspond to the scenes surrounded by a rectangle of the same color. The important scene surrounded by the red rectangle, a scene in which the batter takes a chance, was detected by the PM. Interestingly, the contents of tweets for the scene surrounded by the red rectangle indicate the enthusiasm and expectations of the viewers for this scene. In addition, the important scene surrounded by the blue rectangle is a scene in which the batter hits a home run. The contents of tweets for the scene surrounded by the blue rectangle include the delight and excitement of the viewers watching this important scene. These tweets were posted after the corresponding important scenes. From these results, since there obviously exist time-lags between tweets and their corresponding events, we can confirm that the PM accurately detects these important scenes from this qualitative evaluation.

Finally, we show the results of the Tl-MVAE obtained when parameters λ and *L* of the Poisson distribution are changed in Table 4. The results of each parameter λ express how much the peak of the distribution should be slid. Thus, we can confirm when tweets are posted after corresponding events have occurred. Furthermore, the results obtained when *L* is changed express how much previous events affect the tweets. From the specificity of each parameter, we can confirm that the highest specificity is achieved when λ is three and *L* is 12. In addition, the tweet for the test is posted about every 24 s on average. Therefore, these results suggest that the time-lag between the tweet and its most related event is about 72 s, and events up to 288 s in the past affect the tweets. We therefore consider that the calculation based on the parameters λ and *L* of the distribution is effective for revealing the time-lags.

## 4. Conclusions

In this paper, a new method for the detection of important scenes in baseball videos via the Tl-MVAE is presented. By introducing the consideration of time-lags, the Tl-MVAE can correctly consider the relationships between tweets and videos. Experimental results confirm that the Tl-MVAE realizes the accurate detection of important scenes of baseball videos. Since the immediately previous event strongly influences the tweet and the influence of past events tends to be gradually weakened, we adopt a Poisson distribution for the consideration of time-lags. In future work, we will use this distribution and other distributions in order to verify the optimal distribution for the consideration of time-lags. 

## Figures and Tables

**Table 1:** Details of the baseball video dataset used in our experiment.

Games	Number of Important Scenes	Average Time Length of Important Scenes	Game Time
1	12	2 min 28 s	3 h 23 min
2	14	2 min 31 s	2 h 59 min
3	22	2 min 03 s	3h 32 min
4	18	1 min 58 s	3 h 08 min
5	11	1 min 40 s	2 h 44 min

**Table 2:** Features used in Comparative Methods (CMs) 1-6.

Features	CM1	CM2	CM3	CM4	CM5	CM6
Tweet	✓			✓	✓	
Video		✓		✓		✓
Audio			✓		✓	✓

**Table 3:** Specificity of the detection of important scenes in the proposed method (PM) and CMs1-12.

Games	PM	CM1	CM2	CM3	CM4	CM5	CM6	CM7	CM8 [11]	CM9 [8]	CM10 [15]	CM11 [24]	CM12 [13]
1	0.377	0.350	0.311	0.311	0.350	0.345	0.350	0.345	0.370	0.377	0.360	0.370	0.370
2	0.454	0.363	0.363	0.390	0.390	0.406	0.406	0.363	0.436	0.436	0.406	0.418	0.418
3	0.472	0.449	0.462	0.415	0.449	0.449	0.453	0.453	0.453	0.471	0.462	0.463	0.453
4	0.320	0.301	0.297	0.297	0.301	0.297	0.301	0.297	0.297	0.320	0.308	0.302	0.297
5	0.423	0.365	0.346	0.346	0.409	0.402	0.409	0.365	0.423	0.404	0.421	0.413	0.409
Average	0.409	0.366	0.356	0.352	0.380	0.380	0.384	0.365	0.396	0.402	0.391	0.393	0.389

**Table 4:** Average specificity of the Tl-MVAE for all games when parameters the λ and *L* of the Poisson distribution are changed.

	L=10	L=12	L=14	L=16	L=18	L=20	Average
λ=1	0.396	0.400	0.391	0.387	0.391	0.375	0.390
λ=3	0.403	0.409	0.397	0.392	0.387	0.381	0.395
λ=5	0.391	0.397	0.386	0.381	0.375	0.374	0.384
λ=7	0.377	0.386	0.369	0.364	0.365	0.363	0.371
λ=9	0.364	0.375	0.373	0.365	0.357	0.347	0.364
Average	0.386	0.393	0.383	0.378	0.375	0.368	0.381
